# Modular Soft Sensor Made of Eutectogel and Its Application in Gesture Recognition

**DOI:** 10.3390/bios15060339

**Published:** 2025-05-27

**Authors:** Fengya Fan, Mo Deng, Xi Wei

**Affiliations:** 1 School of Computer Science and Technology, University of Science and Technology of China, No. 96, Jinzhai Road Baohe District, Hefei 230026, China; fanfengya@mail.ustc.edu.cn (F.F.); sa20011167@mail.ustc.edu.cn (M.D.); 2 Department of Biomedical Engineering, School of Instrument Science and Opto-Electronics Engineering, Hefei University of Technology, No. 193, Tunxi Road, Hefei 230009, China

**Keywords:** gel sensor, stretchable sensor, gesture recognition, soft wearable device, modular design

## Abstract

Soft sensors are designed to be flexible, making them ideal for wearable devices as they can conform to the human body during motion, capturing pertinent information effectively. However, once these wearable sensors are constructed, modifying them is not straightforward without undergoing a re-prototyping process. In this study, we introduced a novel design for a modular soft sensor unit (M2SU) that incorporates a short, wire-shaped sensory structure made of eutectogel, with magnetic blocks at both ends. This design facilitates the easy assembly and reversible integration of the sensor directly onto a wearable device in situ. Leveraging the piezoresistive properties of eutectogel and the dual conductive and magnetic characteristics of neodymium magnets, our sensor unit acts as both a sensing element and a modular component. To explore the practical application of M2SUs in wearable sensing, we equipped a glove with 8 M2SUs. We evaluated its performance across three common gesture recognition tasks: numeric keypad typing (Task 1), symbol drawing (Task 2), and uppercase letter writing (Task 3). Employing a 1D convolutional neural network to analyze the collected data, we achieved task-specific accuracies of 80.43% (Top 3: 97.68%) for Task 1, 88.58% (Top 3: 96.13%) for Task 2, and 79.87% (Top 3: 91.59%) for Task 3. These results confirm that our modular soft sensor design can facilitate high-accuracy gesture recognition on wearable devices through straightforward, in situ assembly.

## 1. Introduction

Wearable devices have emerged as promising tools in various domains, ranging from healthcare monitoring to human-computer interaction, where their sensor design plays a crucial role in functionality advancement. Wearable devices using inertial sensors [1,2,3,4], optical sensors [5], or acoustic sensors [6] have been extensively studied. However, these devices are often bulky and do not conform well to the human body, primarily because the sensors themselves are difficult to bend or strain. To address this challenge, flexible sensors based on conductive materials have increasingly emerged [7,8]. Among them, conductive gel, characterized by its piezoresistive properties resulting from the redistribution of internal ions upon mechanical deformation, represents a promising soft sensing material for wearable devices owing to its tunable resistivity, flexibility, stiffness, and biocompatibility. Numerous studies have demonstrated its potential applications in flexible strain and pressure sensors for motion perception in wearable devices [9,10]. However, a few challenges remain unsolved, including the following:

(1) Most gel surfaces are susceptible to environmental contamination and are not suitable for direct exposure to air. Therefore, gel sensors used for gesture recognition should be packed to protect the gel material and prolong its shelf life.
(2) Sensing in conductive gels usually relies on the signal response to mechanical deformation (e.g., stretching, inflation, compression), which may cause displacement between the sensor and the measuring circuit. A connecting method is needed, which can not only support the connectivity between the soft material and the rigid circuit but also remain stable during mechanical deformation.
(3) It is common for gel materials to go through an aging process and gradually lose functionality. The design of the sensor array should allow for the facile replacement of broken sensor units without interfering with the functional ones.
(4) The adhesion force between the gel and the attached interface is rather weak. To secure the mounting, conductive gel sensors are usually embedded into the wearable device as a structurally inseparable part. A reversible mounting/connecting strategy is needed to reduce costs during sensor optimization and achieve fast prototyping.

**Figure 1:**
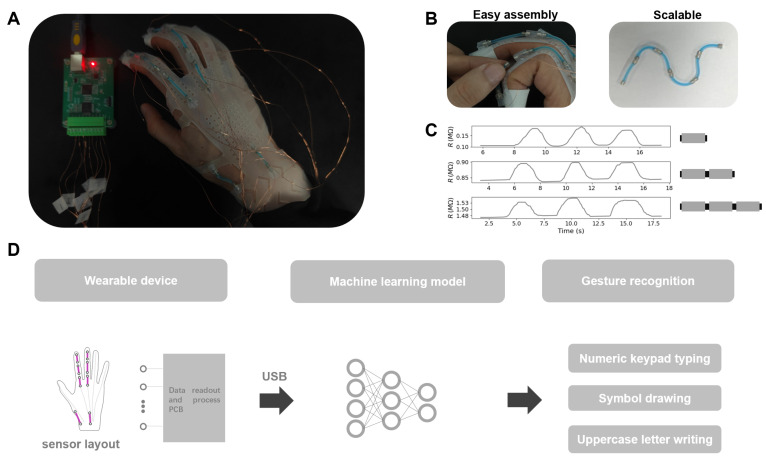
Smart glove based on M2SUs: (**A**) photograph of the smart glove. (**B**) M2SU features easy assembly and scalable series connection of multiple sensors. (**C**) resistance responses under cyclic stretching for 1 M2SU, 2 M2SUs in series, and 3 M2SUs in series. (**D**) data processing workflow of the smart glove.

To address the above-mentioned challenges, we proposed a modular soft sensor unit (M2SU) design using wired-shaped eutectogel with a protective sheath and magnetic ends as the building block to construct a soft sensor array. The sensor’s functionality can be preserved by packing the piezoresistive gel within silicone tubes to protect it from external damage. By using magnets as the sensor’s electrodes and the assembly method, we can achieve secure sensor connections, facile reconfiguration of the array, and reversible sensor mounting on the wearable device. To investigate the perception performance of our proposed design, a silicone glove with sensors attached through magnetic connections was used as a wearable device example to carry out three gesture recognition tasks: numeric keypad typing (0–9), symbol drawing, and uppercase letter writing. A 1D convolutional model was employed to classify the data collected during these tasks. Figure 1A illustrates a photograph of our designed wearable device, Figure 1B demonstrates the M2SU’s features of easy assembly and scalable series connection of multiple sensors. Figure 1C presents the resistance response under strain for varying numbers of serially connected sensors. Figure 1D illustrates the data processing workflow of the smart glove.

To summarize, our paper contributes in the following ways:

(1) We presented a novel design for a modular soft sensor unit (M2SU) described as a short, wire-shaped sensory structure composed of eutectogel packed in a sheath with magnetic blocks at both ends. Compared to existing gel sensors, our proposed sensor is more suitable for complex human-machine interaction scenarios. Additionally, the modular sensor has an excellent strain range, high strain sensitivity, and good durability.
(2) We constructed a silicone glove with 8 M2SUs for hand motion recognition. The sensory array was reversibly mounted on the wearable device through pre-embedded magnetic blocks. This strategy allows for the modification of the sensing module to be independent of the attached device by simply repositioning the sensor layout.
(3) We developed a neural network to analyze the data gathered from the wearable device during three gesture recognition tasks comprising a total of 46 gestures. The network achieved task-specific accuracies of 80.43% (Top 3: 97.68%) for Task 1, 88.58% (Top 3: 96.13%) for Task 2, and 79.87% (Top 3: 91.59%) for Task 3. These results validated the perception performance of M2SUs for gesture recognition.

## 2. Related Works

Here, we present relevant research from two categories: the studies of gesture recognition using flexible sensors, and the current works using gel materials in the realm of wearable perception.

### 2.1. Gesture Recognition with Flexible Sensors

Research on flexible sensors involves advancements in materials, fabrication methods, and structural designs. Fabric-based wearable devices have the capability to recognize the signatures of human grasp [11] and human-environment interactions [12], making them seamlessly integrated into daily life. For instance, the smart strip, composed of three layers of strips with different functions, can utilize touch and stretch as input modalities to interact with virtual content displayed on smart glasses [13]. Electronic tattoos, which are more conformal, can be utilized for movement monitoring and remote control of robots [14]. There are also many efforts to couple the elasticity of soft materials with sensing functions by using polymer composites, e.g., combining elastomers such as Polydimethylsiloxane (PDMS) or Ecoflex as substrates with conductive materials to gain both elasticity and conductivity. For example, PDMSkin [15], encapsulating capillary Ag-PDMS conductive traces in PDMS, was applied in gesture input recognition. Similarly, YSSA [16], made of conductive yarn wound around rubber microfibers and wrapped in a PDMS sleeve, was proven effective in sign-to-speech translation. These studies have demonstrated that the unique features of flexible sensors, such as being lightweight, soft, stretchable, and user-friendly, play an important role in efficient wearable sensing.

Flexible sensors in an array format are commonly used for complex motion sensing [16,17,18]. To customize the layout of sensors in flexible wearable devices, Kim et al. achieved rapid hand tracking and recognition by printing nanomesh receptors directly onto the skin using a portable printing device [19]. Although this approach enables customized sensor layouts through direct printing, it is still time-consuming to implement and requires professional cleaning for removal. FabHandWear, introduced by Paredes et al., offers an innovative system for the end-to-end design and fabrication of customized, functional, self-contained hand wearables [20]. However, once made, this system is limited in its functionality due to irreversible reconfiguration in situ. These methods provide robust tools for implementing customized wearable devices, but the process for reconfiguration is still costly and time-consuming. Therefore, an assembling/implementing method that allows for facile alterations in situ could present new solutions for wearable sensing in multi-task applications.

### 2.2. Wearable Perception with Gel Materials

Gel-based sensors have attracted immense research interest in wearable devices and health-monitoring applications. A conductive hydrogel composed of a polyacrylamide/chitosan hybrid network can be applied as a soft human-motion sensor, providing real-time and accurate detection of both large-scale and small human activities due to its excellent flexibility, puncture resistance, and self-adhesive properties on the skin [21]. The PVA/PA/Gel (PPG) hydrogel, with temperature-triggered tunable mechanics, can reliably adhere to the skin and detect electrophysiological signals under a hot compress, while being easily removed under a cool compress, thus avoiding skin irritation, rubefaction, and pain upon device removal [22]. Conductive polymerizable rotaxane hydrogels can sensitively detect and distinguish both large body motions and subtle muscle movements, and have highly repeatable adhesion to human skin [23]. Sensors containing gels with diverse properties can possess multimodal sensing capabilities. For example, an all-gel multimodal cutaneous sensor composed of PANi-PVC composite gel and PVDF-TrFe gel enables simultaneous single-site monitoring of four biophysical signals (BP, ECG, EMG, and MMG) [24].

Eutectogels, an emerging class of ionic materials developed very recently, use a deep eutectic solvent as their liquid phase, sharing many features with ionic liquids such as high thermal stability and ionic conductivity [25,26]. Xiaowen Xu et al. proposed a high-sensitivity and antifreeze strain sensor by embedding a silver nanowire film into a eutectic gel matrix, which can be used for human motion monitoring and real-time electrocardiogram (ECG) applications [27]. In our previous research, we packed piezoresistive eutectogel in Ecoflex tubes to create soft sensing wires, which facilitated the perception function of a soft gripper [28]. These studies underscore the potential applications of gel sensors in soft sensing and health monitoring.

Despite the above-mentioned merits, there are challenges associated with the practical application of gel materials. For instance, gel materials are prone to fracture, bio-fouling, and degradation due to their soft structural nature. Additionally, the wiring between soft gel sensors and the rigid measurement circuits is difficult, causing instability in the sensing system.

## 3. Fabrication and Characterization of M2SU

### 3.1. Fabrication

**Figure 2:**
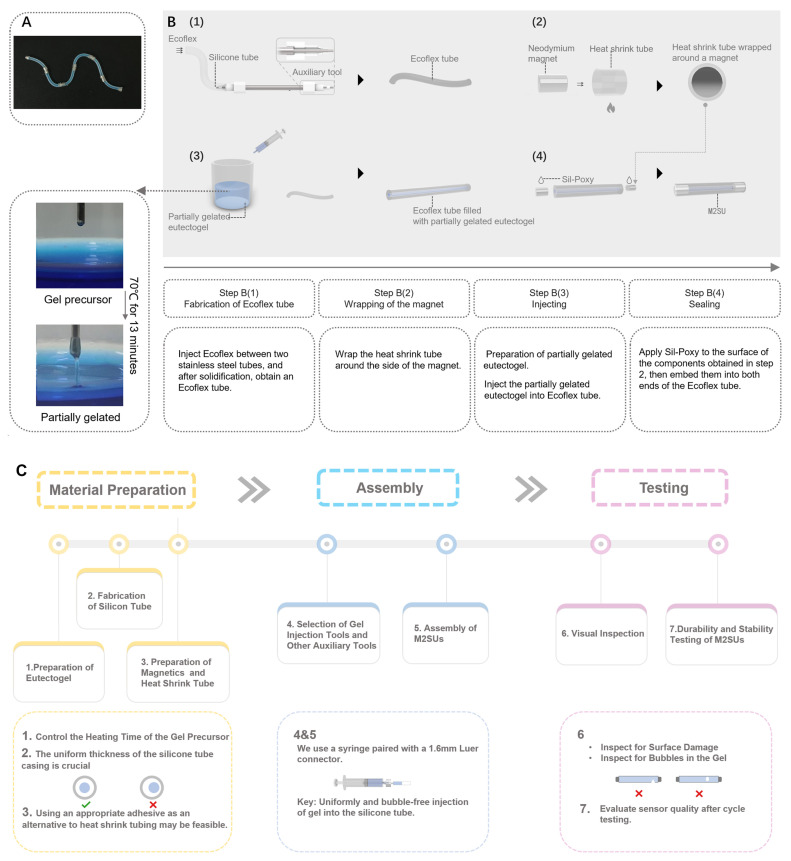
Preparation of the M2SUs: (**A**) optical image of M2SUs. (**B**) schematic diagram of fabricating M2SUs. (**C**) the roadmap for the manufacturing process of M2SU.

Figure 2A is an optical image of a typical M2SUs (with 6 units). We followed a four-step procedure for fabricating the eutectogel sensor (Figure 2B). The eutectogel is transparent; we added blue food dye to enhance its visibility.

- Step 1: The Ecoflex silicone tube was fabricated using a 3D-printed auxiliary tool, which aligned a 2.4 mm inner diameter stainless steel tube with a 1 mm outer diameter stainless steel tube. Ecoflex was then injected between the tubes and allowed to solidify, yielding the silicone tube.
- Step 2: The magnet electrodes (ϕ2mm×3mm) were encased with heat-shrink tube. This procedure was necessary because Sil-Poxy is proficient at bonding polymers and silicone, but it lacks strong adhesion between metal and Ecoflex. Consequently, the sealing of the gel sensor depended on the frictional fit between the magnet and the heat-shrink tube, with the heat-shrink tube adhering to the silicone shell using Sil-Poxy.
- Step 3: The eutectogel used was constructed with a DES (ChCl–EG–urea), zwitterionic sulfobetaine, and Zn(ClO_4_)_2_.The preparation of the eutectogel adhered to methods outlined in a previous publication [29]. Since fully gelated eutectogel proved challenging to inject into slender tubes with a 1 mm inner diameter, we heated the gel precursor to a partially gelated state, facilitating its injection into the silicone tube using a syringe.
- Step 4: Neodymium magnets were used to seal the sensors. At both ends of the sensor, a three-layer structure of magnet, heat-shrink tube, and Ecoflex was tightly bonded to prevent gel leakage.

Figure 2C depicts M2SU’s manufacturing roadmap, incorporating detailed precautions and recommended tools for each processing stage. This visual guide not only assists researchers in comprehending our method to develop improved sensors but also establishes a foundational framework for automated production systems.

### 3.2. Characterization

**Figure 3:**
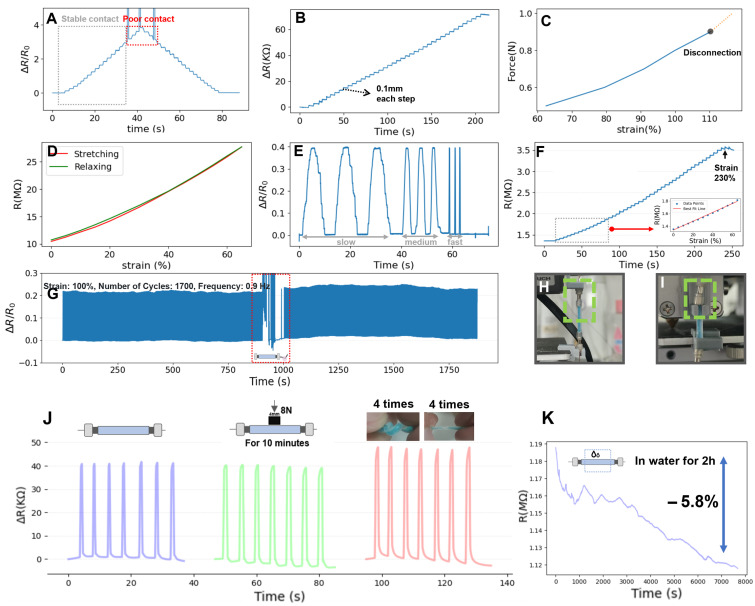
Sensing performance of the M2SU (length: 20 mm): (**A**) Relative resistance responses of the M2SU under stretching from 20 mm to 40 mm in 20 steps. Within the 0–80% strain range, the magnetic force is sufficient to ensure stable connection of the sensor. (**B**) Resistance responses of the M2SU under stretching from 20 mm to 24 mm in 40 steps. (**C**) The tensile force during the stretching process. (**D**) The relative resistance change curve of M2SU during stretching and relaxing in the 0–65% strain range. (**E**) Response curve of the M2SU reaching 60% strain at different strain rates: slow (3.6 s), medium (1.4 s), and fast (0.2 s). (**F**) Resistance change curve of M2SU during 0–230% strain, with linearity $R^{2}=0.995$ in the 0–65% strain range. (**G**) Relative resistance change of M2SU during a 100% strain cycle test at 0.9 Hz. (**H**) Optical image of the test setup showing magnets serving dual functions as mechanical connectors and electrical conductors. The green dotted frame indicates the connection region. (**I**) Optical image of the test setup showing magnets functioning solely as electrical conductors. The green dotted frame indicates the connection region. (**J**) Respond curves under 70% strain cycling after different interferences: initial state, after pressing, and after twisting; (**K**) Resistance change of M2SU after soaking in water (excluding the ends) for 2 h.

The two ends of the M2SU (length: 20 mm) were connected to the electrical testing system (Keithley-2450, Tektronix Inc., Beaverton, OR, USA) via magnets for measurement. The strain range and sensitivity are important indicators of sensors. Figure 3A shows the tensile testing of the sensor, during which it was stretched from 20 mm to 40 mm in 20 steps. Instability in the connection was observed above 80% strain amplitude, which was attributed to insufficient magnetic force to withstand the tension. Figure 3B illustrates the sensor tensile test, during which it was stretched from 20 mm to 24 mm in 40 steps with the strain resolution of the M2SU reaching 0.1 mm. Figure 3C depicts the force generated throughout the sensor’s tensile deformation process, the maximum force that the magnetic connection can withstand is 0.9 N. Figure 3D shows the resistance changes of a 20 mm-long sensor during uniform stretching to 65% strain and subsequent relaxation to its initial state. The maximum hysteresis does not exceed 5%.

In addition to sensitivity and strain range, the consistency of the sensor’s response to strains at different rates is equally critical for sensing performance. Figure 3E shows the response curve of the M2SU reaching 60% strain at different strain rates: slow (3.6 s), medium (1.4 s), and fast (0.2 s). The results are nearly identical at 60% strain for slow and fast rates, suggesting that the sensor has a short response time. In the above strain experiment, the sensors are connected using magnetic force, with the magnets simultaneously functioned as conductive terminals, as shown in Figure 3H. To explore the sensors’ intrinsic strain limit, disregarding magnetic force insufficiency, we conducted an ultimate strain test under the conditions in Figure 3I. Figure 3F shows the resistance change of M2SU during 0–230% strain (excluding magnetic effects). This range meets gesture monitoring requirements and reduces sensor damage risk during human-machine interaction. Notably, the sensor demonstrates excellent linearity (R^2^ = 0.995) within the 0-65% strain range. To investigate the cyclic stability of the M2SU, we tested the sensor’s response to 1700 tensile/release cycles at a 100% strain amplitude, as shown in Figure 3G. The results indicate that the M2SU remained operational after 1700 cycles.

To further investigate the durability and reliability of M2SU in real-world human-machine interaction scenarios, two experiments were conducted: (1) Testing the effects of pressing and twisting on the sensor; (2) The sensor’s waterproof test.

1. Press-Twist Experiment of M2SU. Flexible sensors often experience compression and twisting in human-machine interactions. In the compression test, we applied an 8 N force with a rigid indenter to a 4 mm central region of the sensor for 10 min, followed by a cyclic strain test at 70% strain. Figure 3J shows that the sensor’s initial resistance slightly changed after pressing and stabilized over multiple cycles. In the twisting experiment, the sensor was twisted 8 times, causing a greater relative resistance change compared to the original state but remaining consistent during multiple tensile cycles.
2. Waterproof Experiment of M2SU. We encapsulated the eutectogel with Ecoflex silicone for waterproofing (excluding the ends). Figure 3K shows the sensor’s resistance change during a 2 h water immersion. The resistance decreased by 5.8%, indicating minimal impact from short-term water exposure.

**Figure 4:**
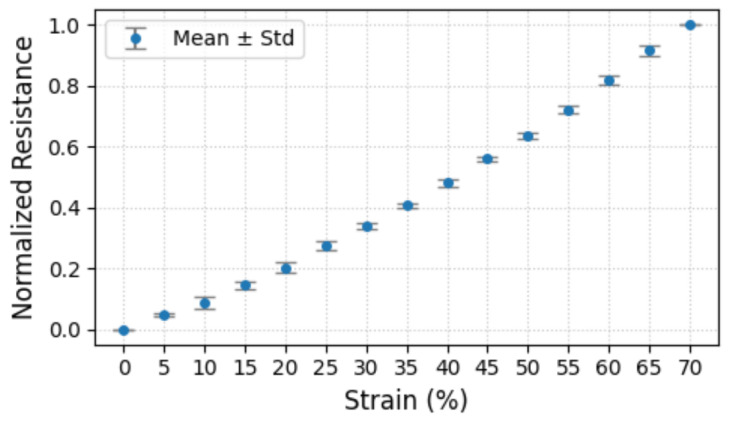
Five M2SU sensors were stretched from 20 mm to 34 mm in 14 steps, with each step increasing by 1 mm. The figure shows the average normalized resistance values of the five sensors under different strain conditions.

For gesture perception, we utilized multiple sensors to monitor joint movements. Consistent sensor outputs under identical strain conditions are essential for reliable measurement. However, initial resistance variations may occur due to manufacturing errors and differential sensor aging. To address this, we implemented min–max normalization of resistance values during strain application, enabling equitable comparison between sensors. In our experiment, five M2SU sensors were stretched from 20 mm to 34 mm in 14 steps, each step being 1 mm. Figure 4 presents the average normalized resistance and standard deviations for these sensors at each strain. The findings confirm that normalization ensures consistent sensor responses to the same strain.

Table 1 presents a comparison of other state-of-the-art flexible strain sensors with M2SU. Based on this comparison, we draw the following conclusions:

1. M2SU exhibits a good strain range and excellent durability. In scenarios involving magnetic connections, the sensor’s 0–80% strain range is well-suited for gesture motion sensing. The 230% strain limit ensures that the sensor is resistant to tensile damage. Furthermore, the 1700 tensile/release cycles at 100% strain have demonstrated M2SU’s outstanding durability.
2. The design of M2SU places greater emphasis on the application of the sensor in human-machine interaction scenarios. With magnetic blocks at both ends, this design facilitates the easy assembly and reversible integration of the sensor directly onto a wearable device in situ. We have investigated the effects of pressing and twisting on M2SU and tested its waterproof capabilities. The results further demonstrate the practical value of M2SU in real-world human-machine interaction scenarios.

## 4. Gesture Recognition Based on M2SUs

**Figure 5:**
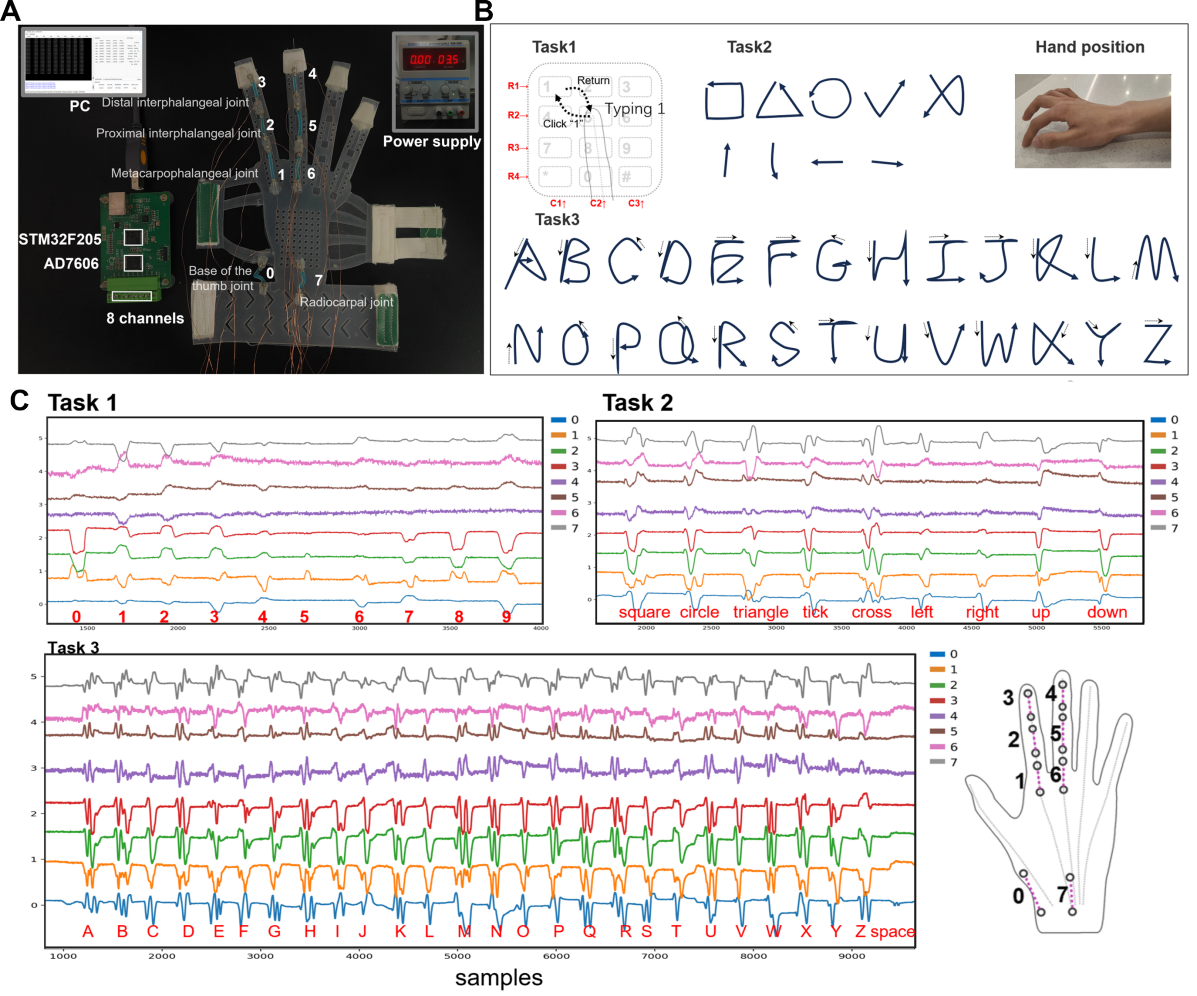
(**A**) The structure of our gesture recognition system. The wearable device transfers data to a computer (PC) via USB, while the sensors are powered by an external power source. The diagram includes the numbering and positions of 8 M2SUs. The microcontroller reads voltage signals through a 16-bit, 8-channel ADC (Analog-to-Digital Converter). (**B**) Gestures of the three tasks. (**C**) Voltage signal when performing gestures.

To investigate the applicability of eutectogel sensors in wearable perception, we developed a silicone glove equipped with 8 M2SUs. In our prototype glove design, the glove’s shell was fabricated using silicone molding and secured to the hand with elastic bands. Sensors are connected to the glove via designated magnetic mounting points on the glove. We deployed 8 M2SUs to capture the movements of the index finger, middle finger, and wrist. Our gesture recognition system is illustrated in Figure 5A.

We selected three tasks: numeric keypad typing (Task 1), symbol drawing (Task 2), and uppercase letter writing (Task 3). Task 1 exhibits a relatively shorter gesture duration compared to Tasks 2 and 3, facilitating a concurrent assessment of the device’s capacity to distinguish between short and prolonged actions. The gestures across these three tasks adhere to habitual patterns, while the recognized content corresponds to common text input. Ultimately, we employed our designed neural network to classify the acquired data.

### 4.1. Three Gesture Tasks

Wearable devices using strain sensors can capture time-series signals, while variations in writing strokes for the same symbol can manifest significant differences in signals. In our experiment, we defined specific gesture patterns for the three tasks to reduce the manual interference. The initial state of the hand before performing a gesture is depicted in Figure 5B. The gestures included in the experiment and their execution methods are as follows:

- Task 1: During the performance of the gestures, the forearm remains stationary. The index finger initiates the gesture from the number ‘5’ position, then moves toward the designated number and clicks on it. Finally, the index finger returns to the initial position. In the actual execution process, no physical keyboard guides the finger’s direction. For instance, participants rely on their proprioception to guide the finger to click on number ‘1’ and then return to ‘5’.
- Task 2 and 3: The palm’s position remains consistent with Task 1. In Task 2, the gestures entail drawing a square, a triangle, a circle, a tick, a cross, and movements in the directions of up, down, left, and right. For Task 3, the gestures involve writing all 26 uppercase letters and recognizing the ‘space’. Upon completing each gesture, the fingertip of the index finger returns to the initial position. The motion of tapping the desktop with the thumb represents the ‘space’ gesture.

### 4.2. Dataset

We recruited nine volunteers (three males, six females, average age 24 years, range 21–27 years) to sit in front of a table and, in response to task prompts displayed on a screen, execute clicks or writing gestures. Each participant wore the device on his(her) right hand, utilizing the index finger for both clicking and drawing gestures. We developed a dedicated program to indicate the next number to click or letter to write. During Task 1 data collection, each round consisted of 80 samples, with each number appearing 8 times and prompts occurring every 2.6 s. For Task 2 data collection, each round comprised 54 samples, with each gesture appearing 6 times and prompts occurring every 4 s. In Task 3 data collection, each round comprised 108 samples, with each character appearing 4 times and prompts occurring every 4 s. Prompt intervals and sample counts for each round were adjusted empirically based on the participants’ reaction times and attention span. We conducted three rounds for each task, resulting in 2160 samples for Task 1, 1458 samples for Task 2, and 2916 samples for Task 3. The sampling rate was set to 100 Hz, and part of the collected voltage signal when performing gestures is shown in Figure 5C.

### 4.3. Data Processing

**Figure 6:**
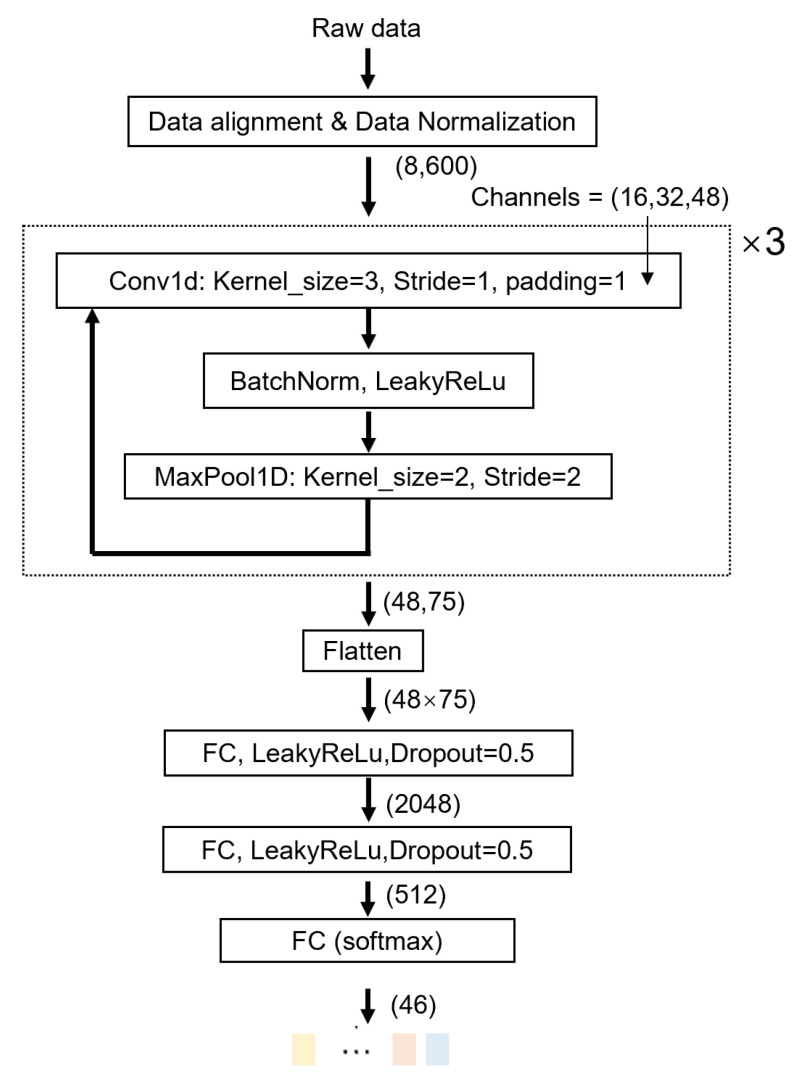
Data Processing Pipeline: Our primary input consists of continuous streams from 8 M2SUs. The three tasks have different gesture durations (Task 1: 120 sampling points; Task 2: 300 sampling points; Task 3: 260 sampling points). The data preprocessing steps include min–max normalization. The gesture data samples for all tasks are resampled to the same length using nearest neighbor interpolation. Our architecture comprises three blocks of convolutional layers followed by three final linear layers, culminating in a softmax activation function that produces a probability distribution for all gestures (n = 46).

We utilized the duration of the longest gesture in each task as the time window size for that task, resulting in time window sizes of 1200 ms for Task 1, 3000 ms for Task 2, and 2600 ms for Task 3. For each captured data sample, we applied min–max normalization. To ensure that the data samples for gestures across the three tasks have consistent lengths, we applied nearest neighbor interpolation to resample the data of the three tasks to a length of 600. Consequently, the input signal size for each gesture is (8, 600). We employed a 1D convolutional neural network for feature extraction with a learning rate of 0.001. The network was trained for a total of 50 epochs, with the data partitioned into training and test sets in a 6:4 ratio. The specific network model is illustrated in Figure 6.

We introduced three data augmentation techniques during model training to increase the number of training samples [37]. These techniques include the following: (1) jittering, to simulate minor fluctuations in gesture data, with the jittering factor $s∼N(1,0.1^{2})$; (2) scaling, to simulate different gesture strength, with the scaling factor $s∼N(1,0.1^{2})$; (3) time-warping, to simulate gesture temporal variance, with two interpolation knots and warping randomness $w∼N\left({1,0.1}^{2}\right),w\in \left[0,2\right]$.

### 4.4. Evaluation

#### 4.4.1. Gesture Recognition Performance

**Figure 7:**
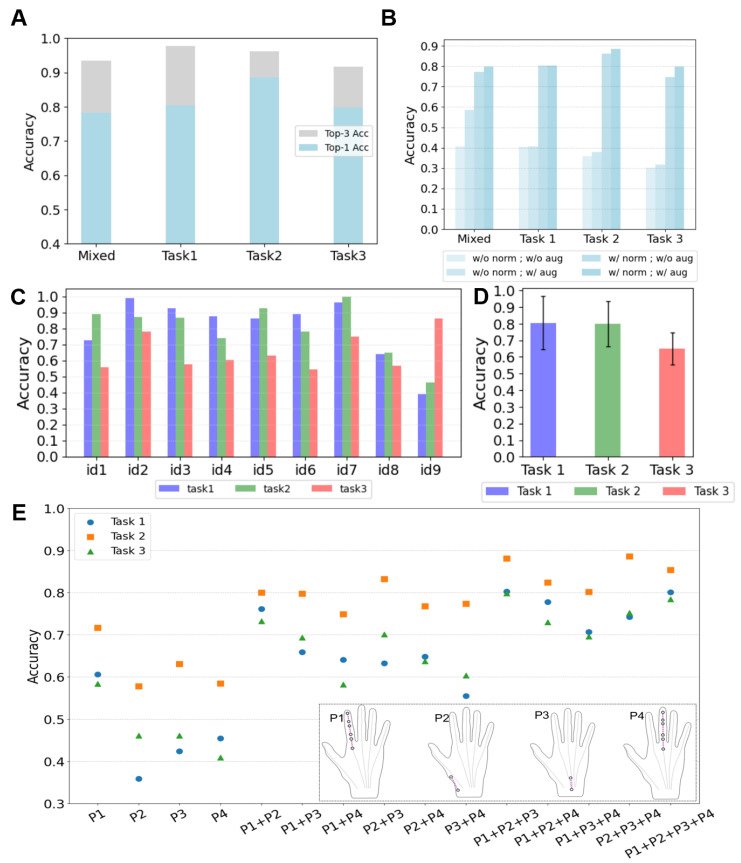
(**A**) The Top-1 and Top-3 recognition accuracy for the three tasks when trained separately, and the recognition accuracy when all tasks are trained together. (**B**) Gesture recognition results on different datasets under various data processing conditions. (**C**) The accuracies of gesture recognition when each individual’s data are used to create separate datasets. (**D**) Average accuracy across all participants for each task with 95% confidence intervals illustrated. (**E**) Recognition accuracy using different sensor layouts: P1—Index finger (gesture finger), P2—Base of the thumb joint, P3—Radiocarpal joint, P4—Middle finger.

**Figure A1:**
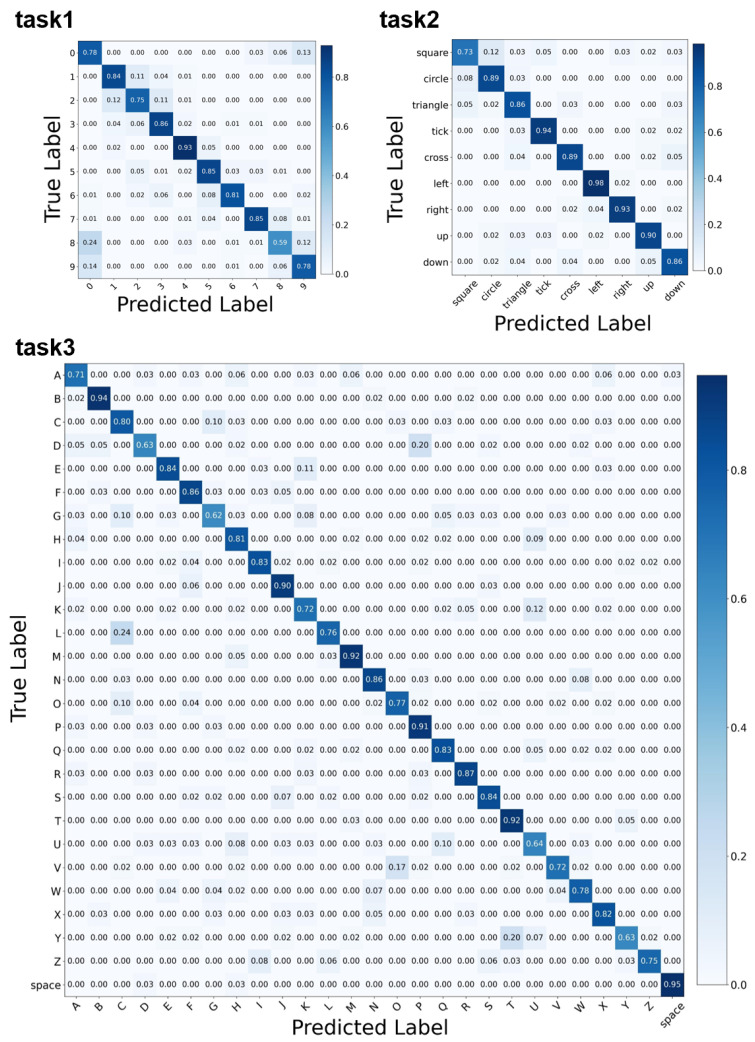
The confusion matrices for the three tasks respectively.

**Figure A2:**
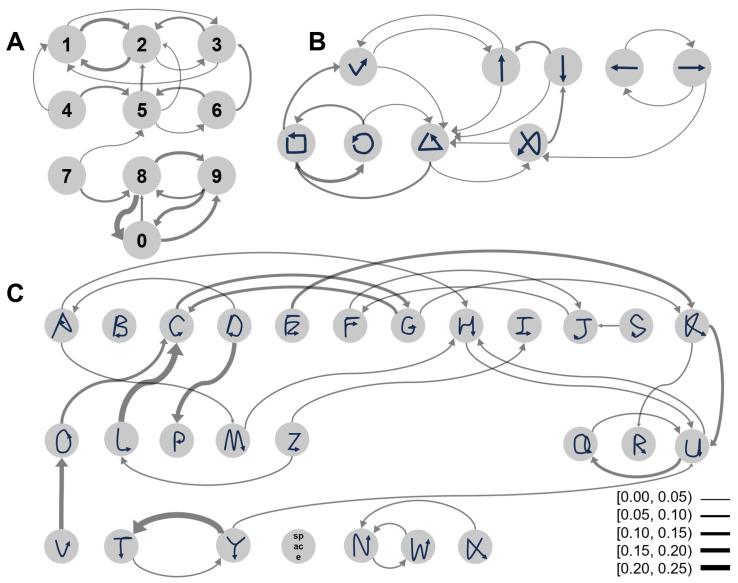
Confusion Map Highlighting Real Gestures and Their Two Most Easily Confused Counterparts. Each arrow originates from the actual gesture and points to one of the two gestures most frequently confused with it. The thickness of the lines indicates the confusion ratio, categorized into five distinct levels. (**A**) Confusion map for Task 1. (**B**) Confusion map for Task 2. (**C**) Confusion map for Task 3, with arrows in the range [0, 0.05) excluded to accentuate the most easily confused gesture pairs.

We constructed separate multi-person datasets for each task and a mixed dataset that included all gestures from all participants. Figure 7A shows the gesture prediction results after training the model using these four datasets separately. To explore the differences in individual participants’ data, we created single-person datasets for each participant and task. Figure 7C shows the classification results of these single-person single-task datasets. Given the experimental results, we draw the following conclusions:

1. Classification errors primarily occur between similar gestures. Figure A1 shows the confusion matrices of the recognition results of the three tasks. To provide a more intuitive visualization of the similarities between confusing gestures, we developed a confusion map based on the confusion matrix. In Figure A2, each gesture is linked to the two gestures most frequently confused with it via arrows. The confusion map reveals that in Task 1, gestures for adjacent numbers (“1–2–3” and “8–9–0”) are particularly prone to confusion. This issue arises because participants rely on proprioceptive feedback for number selection, which impedes precise finger positioning. In Tasks 2 and 3, gesture confusion is primarily due to the inherent similarity of symbols (e.g., “square” vs. “circle”, “C” vs. “L”) and the requirement for gestures to return to the starting position. While this requirement facilitates gesture segmentation, it introduces additional movements, thereby making dissimilar gestures, such as “V” and “O”, appear more similar.
2. In the mixed-task recognition scenario, the accuracy for each task is lower than that achieved when training each task separately. When training with a mixed dataset of all three tasks, the recognition accuracies for each task were 78.47% (Task 1), 85.45% (Task 2), and 74.23% (Task 3). In contrast, accuracies achieved by training each task separately were 80.43% (Task 1), 88.58% (Task 2), and 79.87% (Task 3). More gestures tend to lead to higher gesture confusion.
3. Recognition results from individual datasets varied significantly among participants (Figure 7C). Figure 7D shows the average accuracy for each task across all participants and illustrates the 95% confidence intervals. For Task 1, the best accuracy was 98.75% (id2) and the worst was 38.74% (id9); for Task 2, the best was 100% (id7) and the worst was 46.29% (id9); for Task 3, the best was 86.11% (id9) and the worst was 54.20% (id6). On average, data collection took 50 min per participant. Participants id2 and id7 had 20 min more practice time than others, which contributed to their superior results.

#### 4.4.2. Sensors Layout Evaluation

To investigate the impact of different sensor layouts on the recognition accuracy of the three tasks, we divided the sensor layout into four regions: gesture finger (P1), base of the thumb joint (P2), radiocarpal joint (P3), and middle finger (P4). The sensors from four locations can form fifteen combinations. We separately trained the data corresponding to these 15 combinations and obtained the results as shown in Figure 7D. For the data corresponding to the sensors not used, we replaced them with random noise. Based on the experimental results, we draw the following conclusions:

- Different tasks may have different optimal sensor layouts. Task 2 achieved its highest recognition accuracy (88.58%) with sensors P2 + P3 + P4. Tasks 1 and 3 reached their optimal accuracies (80.43% and 79.87%, respectively) with sensors P1 + P2 + P3.
- Utilizing movement patterns caused by complex hand bio-mechanics in a non-gesturing finger (P4) to achieve gesture recognition is possible. During gesture data collection, the middle finger (P4) was unconstrained and moved according to participants’ habits. For Task 2, the accuracy reached 88.58% with P2 + P3 + P4, and 88.12% with P1 + P2 + P3. Thus, sensors on P1 and P4 are equally important for Task 2.
- Adding more sensors does not always enhance recognition accuracy. With the P1 + P2 + P3 layout, Task 2 and Task 3 achieved accuracies of 88.12% and 79.87%, respectively. Adding P4 sensors to this layout reduced the accuracies to 85.35% for Task 2 and 78.43% for Task 3.

### 4.5. Ablation Study

We combined four data processing strategies based on the presence or absence of min–max normalization and data augmentation. We conducted gesture recognition experiments under these four data processing strategies. Figure 7B shows the conditions and corresponding results. In the mixed tasks case, min–max normalization increased accuracy from 40.56% to 77.34%. With data augmentation, accuracy reached 79.87%.

When tested on single-task datasets, both min–max normalization and data augmentation improved gesture recognition accuracy. By scaling data to [0, 1], min–max normalization significantly enhanced accuracy, while data augmentation further improved it by increasing the number of training samples.

## 5. Discussion

Here, we discussed the limitations in M2SUs and its future potential in related applications.

### 5.1. Limitations

In this paper, we presented the fabrication method for eutectogel sensors, constructed a glove incorporating eight sensors as an exemplary wearable device, and investigated the sensing performance through gesture recognition. Throughout this endeavor, several technical obstacles emerged. Our sensor fabrication process predominantly relies on manual methods, making it challenging to control batch variations. Mass fabrication requires solving many process issues to ensure sensor uniformity and reliability. Moreover, due to the natural aging process of the gel material, the shelf life of the M2SU is still limited. When operating under a 3.7 V DC power supply with an average daily usage of 2 h, the sensor maintains stable functionality for 25 days. However, our proposed modular design addresses potential performance degradation by enabling rapid replacement of individual faulty sensors, thereby ensuring long-term device stability.

### 5.2. Potential Application

The M2SU unit proposed in this study can serve as a fundamental building block for a versatile optimization strategy during prototyping. The irreversible connecting strategy among sensing units offers fast prototyping with easy alteration of sensor arrays for specific tasks. We conclude that the gel sensors developed in this study have potential for diverse sensing applications, such as monitoring individuals during their daily activities and controlling home appliances or various equipment, extending beyond gesture recognition as detailed herein, through wearable devices and other interactive interfaces. The proposed strategy can also be optimized and applied to other soft sensing materials for further advances and customized designs.

## 6. Conclusions

In this study, we present a novel approach to building a modular soft sensor unit (M2SU). The short, wire-shaped sensory structure made of eutectogel, with magnetic blocks at both ends, facilitates the reversible integration of the sensor directly onto a wearable device in situ. The M2SU exhibits excellent piezoresistive response to motion-induced stretching and flexibility in reconfiguring sensor layouts in situ through magnetic connections without complicated modification. To explore the practical application of M2SUs in wearable sensing, we equipped a glove with magnetic mounting points. This allowed for the quick and easy assembly of M2SUs using magnetic connections. We evaluated its performance across three common gesture recognition tasks: numeric keypad typing (Task 1), symbol drawing (Task 2), and uppercase letter writing (Task 3). By employing a 1D convolutional neural network to analyze the gesture data, we achieved task-specific accuracies of 80.43% (Top 3: 97.68%) for Task 1, 88.58% (Top 3: 96.13%) for Task 2, and 79.87% (Top 3: 91.59%) for Task 3. We confirm that our modular soft sensor design facilitates high-accuracy gesture recognition on wearable devices through straightforward, in situ assembly. The inherent reconfigurability of this approach presents a versatile optimization strategy during prototyping and confers the capability to alternate sensor arrays with ease for specific tasks.

## Figures and Tables

**Table 1:** Comparison of recent works on piezoelectric and piezoresistive sensors, assessing their sensing range, gauge factor, hysteresis, performance in cyclic testing, material constituents, and the applications showcased, along with a detailed examination of their highlighted sensor characteristics.

Ref.	Materials	Sensing Range (%)	Gauge Factor	Hysteresis	Cycle Test (Cycles)	Characteristics	Applications
[16]	Stainless steel, Rubber, Polyester, PDMS	90	NA	NA	6000 cycles (0–70%)	Low cost; Skin mountability	11 Sign Language Gestures
[30]	PDMS, EGaIn	70	NA	low hysteresis	NA	AC-enhanced Liquid Metal Sensors	Knuckle Flexion Detection, Respiration Tracking
[31]	Fluoroelastomer, EGaIn Nanoparticles	1170	NA	NA	600 cycles (0–100%)	Highly conductive and stretchy; Resistance change of only 4% at 200% strain	Glove/Fabric Integration for Wearable Sensors; Self-powered wearable sensors
[32]	Polyester-Rubber Fabric, PEDOT:PSS	45	4.5	2%	10,000 cycles (0–30%)	Good Linearity (0.98); High Sensitivity (GF = 4.5); Low Hysteresis (2%)	Finger Movement Monitoring
[33]	Ga Liquid Metal, Acrylamide, NaCl, Ammonium Persulfate, Glycerol	0.1–1000	1.2 (strain range of 0–100%) 5.8 (strain range of 100–1000%)	No hysteresis phenomenon observed	4000 cycles (0–10% strain)	Wide temperature range (−20 °C to 100 °C); Low strain detection limit (0.1%); Self-healing abilities	Detect finger bending
[34]	VSNP-PAM hydrogel, PpyNWs, d-Ti_3_C_2_T_x_ MXene layers	2800	16.9 in the x direction and 11.2 in the y direction at 1500% strain	≤10%	1000 cycles (0–800% strain, 0.12 Hz)	Tunable sensing mechanisms: tension sensing capabilities and capacity sensing capabilities (using two strips of MXene-PpyNW-VSNP-PAM stretchable e-skins)	Monitoring stretching motions in multiple dimensions, tactile pressure and proximity sensing.
[35]	Spandex, PDMS, Au, Polyacrylonitrile NFs	0.005–155	NA	NA	>35,000 cycles (0–10% strain, 0.7 Hz)	Low hysteresis and high stability during extensive use and washing cycles	Accurate detection of 48 static and 50 dynamic gestures; Typing on a random surface such as a mock keyboard; Recognition of objects from grasp pose and forces
[36]	Composite organohydrogels	1350	3.19	NA	800 cycles (0–50% strain)	Self-healing; Wide temperature detection range (−40 °C to 60 °C); High sensitivity: 0–500% (GF = 3.19)	Detect human movements such as finger bending, wrist bending, and facial movements
this work	Eutectogel, Ecoflex 0010, Neodymium magnets, etc.	80 (connect by magnetic), 230 (maximum strain range)	3.4 (The Gauge Factor decreases with the aging of the eutectogel)	≤5%	1700 cycles (0–100% strain, 0.9 Hz)	Modular design for HCI; Good linearity (strain 0–65%, $R^{2}\approx 0.995$); Strain sensitive (sensor length: 20 mm, resolution: 0.1 mm); Low cost; High Reliability: Can be pressed, twisted, and waterproof (except for the magnet end)	Three types of dynamic input gestures mentioned in the paper

## Data Availability

All research data supporting this study, including gesture capture datasets, manufacturing tool 3D models, and glove construction material specifications, are publicly available in the M2SU repository at https://github.com/d2cd/M2SU, accessed on 10 April 2025.

## Abbreviations

The following abbreviations are used in this manuscript:

M2SU	modular soft sensor unit
